# Advances in molecular breeding of medicinal plants

**DOI:** 10.1186/s43897-026-00252-9

**Published:** 2026-06-12

**Authors:** Yaqi Wang, Xinlei Jia, Chao Sun, Wansheng Chen, Ying Xiao

**Affiliations:** 1 State Key Laboratory of Discovery and Utilization of Functional Components in Traditional Chinese Medicine, Institute of Chinese Materia Medica, Shanghai University of Traditional Chinese Medicine, 1200 Cailun Road, Pudong New Area, Shanghai, 201203 China; 2 State Key Laboratory of Discovery and Utilization of Functional Components in Traditional Chinese Medicine, Natural Products Research Center of Guizhou Province, Guizhou Medical University, 3491 Gaohai Road, Baiyun District, Guiyang, Guizhou 550014 China; 3 Department of Pharmacy, Changzheng Hospital, Second Military Medical University, 415 Fengyang Road, Huangpu District, Shanghai, 200003 China; 4 State Key Laboratory for Quality Ensurance and Sustainable Use of Dao-di Herbs, China Academy of Chinese Medical Sciences, Beijing, 100700 China

**Keywords:** Medicinal plants, Molecular breeding, Molecular marker-assisted breeding, Genetic engineering breeding, Molecular design breeding

## Abstract

Medicinal plants are rich in bioactive constituents and are extensively utilized in the healthcare sector, holding significant value for both industrial and daily applications. In the context of economic development and escalating environmental challenges, there is an increasing societal demand for medicinal plants with superior quality and enhanced environmental adaptability. Molecular breeding is emerging as a pivotal strategy for the genetic improvement of medicinal plants. Given that traditional breeding methods are often constrained by low efficiency and lengthy developmental cycles, the necessity for innovative approaches is underscored. Advancements in modern molecular breeding technologies, such as marker-assisted breeding, genetic engineering, and molecular design breeding, have revolutionized the paradigm of medicinal plant breeding. This article reviews the research progress in molecular breeding of medicinal plants, encompassing its characteristics, advantages, technical classifications and applications. Based on these advancements, we discuss the major challenges and future prospects in this field. It is evident that the rapid evolution of molecular breeding holds substantial potential for facilitating plant genetic improvement and effectively addressing the growing societal demands for high-quality medicinal plants.

## Introduction

Medicinal plants are rich in a diverse array of bioactive compounds (e.g., glycosides, flavonoids, terpenoids, alkaloids, and phenolics), serving not only as vital resources for the prevention and treatment of diseases but also as key sources of ingredients for dietary supplements, pesticides, veterinary drugs, and oral care products (Świątek and Adamska-Szewczyk 2024). Over the past fifteen years, driven by population growth and rising public health awareness, the global demand for medicinal plants has surged. However, exacerbated by overexploitation and environmental degradation, wild medicinal plant resources are facing the depletion crisis. To alleviate this pressure, artificial cultivation has emerged as the primary solution. Currently, approximately 30% of medicinal plant species have been domesticated, contributing to over 70% of the total supply of medicinal materials in the market (Qian et al. 2020). Nevertheless, artificial cultivation often triggers a series of challenges, including reduced accumulation of bioactive compounds, continuous cropping obstacles, pest and disease infestations, genetic erosion of cultivars, and high labor intensity.

To overcome these cultivation bottlenecks and satisfy market demands, it is imperative to accelerate the development of high-quality germplasm. In this context, molecular breeding emerges as a powerful engine, offering unprecedented opportunities to accelerate the breeding of superior medicinal plant varieties. Molecular breeding integrates modern biotechnology into classical genetic breeding, with its core lying in the precise selection and modification of genotypes via molecular markers and genome editing. Within this framework, genome sequencing and high-throughput genotyping have enriched the toolkit of quantitative genetics, enabling deeper analysis of genetic variation in natural populations and precise selection based on genomic breeding values (Du et al. 2025). Specifically, marker-assisted selection (MAS) enables the accurate identification of molecular markers linked to desirable traits, thereby achieving the efficient screening of superior varieties and effectively mitigating the risk of genetic erosion. Meanwhile, genetic engineering allows for the introduction of novel alleles or the precise knockout of deleterious genes, demonstrating immense potential in elevating the levels of specific active compounds and enhancing biotic and abiotic stress resistance (e.g., pest, disease, drought, and waterlogging resistance). Looking ahead, the deep convergence of big data applications, information technology, artificial intelligence (AI), and cutting-edge biotechnologies will make breeding strategies more precise and efficient, facilitating the optimal pyramiding of ideal genetic variations (Wallace et al. 2018; Kuriakose et al. 2020). This review systematically elaborates on the progress made in the past fifteen years regarding the core characteristics and technological advantages of molecular breeding in medicinal plants, highlights their practical applications, and provides insights into the current challenges and future developmental trajectories.

## Characteristics and advantages of molecular breeding

Conventional breeding methods, such as hybridization, mutagenesis, and polyploid breeding, have been used to introduce desired traits and enhance the availability of plant genetic resources (Ahmar et al. 2020). Traditionally, these methods depend predominantly on agronomic selection, and nowadays they also place great emphasis on physiological and biochemical indicators, especially for medicinal plants whose primary breeding objectives center on bioactive compounds. However, the overall process of conventional breeding is severely hindered by a long breeding cycle, heavy workload, and low efficiency. Specifically, the efficiency of agronomic and biochemical selection is increasingly recognized as a critical bottleneck limiting genetic improvement. Many medicinal species, such as *Panax ginseng*, exhibit prolonged growth cycles, wherein the accumulation of active ingredients (e.g., ginsenosides) typically peaks only after several years. Under conventional selection, germplasm screening is constrained by the necessity of waiting until plant maturation to harvest samples for extraction and analysis to determine active compound content. This requirement not only prolongs the breeding cycle and exacerbates the already heavy workload, but also severely restricts the scale and efficiency of screening. Furthermore, the biosynthesis of these bioactive components is significantly modulated by environmental factors, resulting in strong genotype-by-environment interactions. Consequently, agronomic and biochemical performances can be significantly influenced by the environment, masking true genetic potential, impeding the accurate identification of individuals harboring superior alleles, and compromising the stability of breeding outcomes.

In contrast, the elucidation of gene function underpins molecular breeding. This paradigm necessitates precise DNA sequence information, particularly markers associated with target traits, shifting the focus toward genotype-driven selection and modification (Zhao et al. 2019). Breeders can leverage key genes regulating target traits, such as bioactive ingredients and disease resistance, as molecular markers for genotyping using minute amounts of DNA. This approach can be implemented at the seedling stage, effectively circumventing the constraints imposed by the prolonged growth cycles of medicinal plants. It enables non-destructive, high-throughput early screening, effectively achieving “juvenile prediction”, thereby significantly enhancing overall efficiency and accelerating the breeding cycle. Consequently, breeders can precisely identify individuals genuinely harboring superior alleles, thereby enhancing the genetic stability of medicinal plants. Furthermore, genetic engineering techniques, encompassing transgenics and genome editing, can overcome interspecific reproductive barriers. Unlike the random mutation characteristic of traditional mutagenesis, these methods achieve precise, targeted trait improvement. These technologies facilitate the introgression of desirable genes (e.g., pest and disease resistance genes) into medicinal plants or enable precise genome editing, thereby fostering the pyramiding of alleles associated with favorable traits (Xia et al. 2019). By utilizing tools such as genomics, transcriptomics, proteomics, metabolomics, and phenomics, molecular breeding enables large-scale genetic analysis and breeding experiments (Xu 2014). For instance, metabolomics can accurately profile active compounds in medicinal plants using high-resolution mass spectrometry (HRMS) and nuclear magnetic resonance (NMR). By integrating metabolite abundance with gene expression data, researchers can identify significantly correlated metabolite-gene association modules, thereby mining key regulatory genes. Obviously, molecular breeding facilitates the precise manipulation of target traits, significantly augmenting the efficiency of genetic variation discovery, creation, and recombination (Du et al. 2025). Meanwhile, molecular breeding significantly reduces land requirements compared to traditional methods. Data reveal that assessing 20,000 combinations necessitates over 8 × 10^4^ m^2^ of land via conventional breeding, whereas molecular breeding demands merely 1 × 10^4^ to 1.3 × 10^4^ m^2^ (EqualOcean Intelligence 2023). Currently, molecular breeding is increasingly demonstrating its research advantages in cultivating medicinal plant varieties.

## Classification and applications of molecular breeding in medicinal plants

With continuous research advancements, molecular breeding technologies have become increasingly integral to the cultivation of medicinal plants. This field encompasses three primary pillars: molecular marker-assisted breeding, genetic engineering breeding, and molecular design breeding. The following sections elucidate the methodologies underlying these techniques and discuss their specific applications and progress in the context of medicinal plant breeding.

### Molecular marker-assisted breeding

**Fig. 1:**
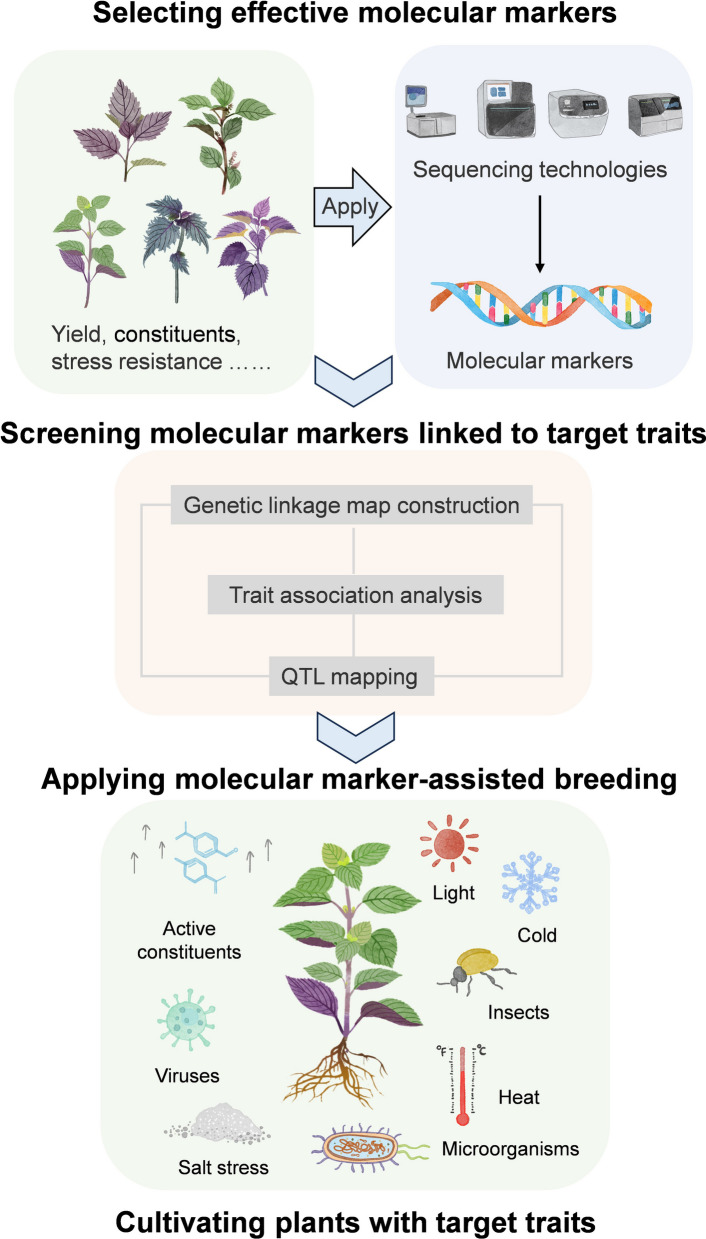
Schematic diagram of the research strategies for molecular marker-assisted breeding in medicinal plants

Molecular marker-assisted breeding utilizes DNA molecular markers linked to target traits for genotype screening, thereby significantly enhancing breeding efficiency (Fig. 1). This methodology encompasses diverse approaches, including marker-assisted backcrossing, marker-assisted recurrent selection, and genomic selection. Currently, it stands as the most widely applied strategy in the molecular breeding of medicinal plants, effectively facilitating the transition from phenotypic selection to genotypic selection.

#### Precise selection assisted by molecular markers

Molecular markers are widely employed in the construction of genetic linkage maps and quantitative trait loci (QTL) analysis to delineate the relationships between genomic regions and their corresponding traits, as well as to analyze genetic diversity. On this basis, superior germplasm can be identified and selected, thereby facilitating variety improvement and the development of new cultivars. Based on analytical methodologies, molecular markers are primarily categorized into three types (Lin et al. 2022, Garrido-Cardenas et al. 2018): (1) those based on Southern blot hybridization (Lander and Botstein 1989), namely restriction fragment length polymorphism (RFLP); (2) polymerase chain reaction (PCR)-based markers (O’Hanlon et al. 2000), which encompass a large number of molecular markers, including random amplified polymorphic DNA (RAPD), sequence-related amplification polymorphism (SRAP), target region amplification polymorphism (TRAP), amplified fragment length polymorphism (AFLP), simple sequence repeat (SSR), and inter-simple sequence repeat (ISSR), cleaved amplified polymorphic sequence (CAPS), among others; (3) markers based on sequencing and DNA microarray technologies (Ganal et al. 2009), such as single nucleotide polymorphism (SNP). Different molecular markers are utilized for the detection and amplification of specific DNA fragments (Dheer et al. 2020). Table 1 outlines the advantages and disadvantages of primary molecular markers and their applications in medicinal plants.

**Table 1:** Advantages and disadvantages of primary molecular markers and their application in medicinal plants

Molecular marker	Acronym	Advantages	Disadvantages	Applications
Restriction fragment length polymorphism	RFLP	Stable results, good reproducibility	Insufficient security, require relatively large quantities of high-quality DNA, require DNA sequence information, long period	*Adenostemma lavenia* (L.) Kuntze (Wu et al. 2022); *Arctium lappa* L. (Hang et al. 2024); *Cirsium japonicum Fisch. ex DC.* (Zhang et al. 2023b)
Random amplified polymorphic DNA	RAPD	Simple operation, low amounts of DNA, the primers are universal, low cost, high polymorphism	Poor repeatability, dominant inheritance, unable to distinguish dominant homozygotes from heterozygotes in F_2_ generation	*Panax ginseng* C. A. Mey. (Ma et al. 1999); *Rubus chingii* Hu (Chen et al. 2022); *Erythropalum scandens* Bl. (Zhao et al. 2024)
Sequence-related amplification polymorphism	SRAP	Simple operation, good reproducibility, lower cost, easy to sequence, no prior sequence information required	Unable to distinguish dominant homozygotes from heterozygotes, require high quality DNA templates, primer design depends on GC content	*Pyrostegia venusta* (Ker Gawl.) Miers (Gavilan et al. 2022); *Clinacanthus nutans* (Burm. f.) Lindau (Chiangchin et al. 2023); *Saussurea medusa* (Wang et al. 2023a)
Target region amplification polymorphism	TRAP	Simple operation, good reproducibility, high reliability, easy to sequence	Unable to distinguish dominant homozygotes from heterozygotes, require high quality DNA templates, primer design depends on cDNA/EST sequences	*Liriope muscari* (Zhang and Chen 2010); *Dendrobium officinale* (Liu et al. 2016); *Magnolia officinalis* (Li et al. 2022)
Amplified fragment length polymorphism	AFLP	Quick, high reliability, high polymorphism	High cost, require high quality DNA templates, high requirements for PCR condition	*Striga asiatica* (L.) O. Kuntze (Hu et al. 2023); *Toona sinensis* (A.Juss.) Roem. (Zhao 2023); *Lycium chinense* Mill. (Zhang et al. 2023a)
Simple repeated sequence	SSR	Good reproducibility, high polymorphism, simple operation, high reliability, high variability, ability to identify homozygous and heterozygous	Difficulties in primer development, high cost	*Cannabis sativa *L. (Li et al. 2023b); *Xanthoceras sorbifolium* Bunge (Li et al. 2024); *Juglans regia* L. (Liu et al. 2024)
Inter-simple sequence repeat	ISSR	Simple operation, low cost, good reproducibility, universal primers, good stability	PCR conditions need to be explored, unable to distinguish dominant homozygotes from heterozygotes in F_2_ generation	*Pueraria lobata* (willd.) Ohwi (Yuan et al. 2017); *Ginkgo biloba* L. (Ge et al. 2003); *Atractylodes chinensis*(DC.) Koidz. (Jiang et al. 2021)
Cleaved amplified polymorphic sequence	CAPS	High polymorphism, simple operation, good reproducibility, high reliability, low cost, low DNA consumption	Require high quality DNA templates, limited polymorphic resolution, low throughput	Juglans cinerea L. (Ebrahimi et al. 2025); *Tetrastigma hemsleyanum* Diels & Gilg (Peng 2016); *Corchorus capsularis* L. (Tao et al. 2020)
Single nucleotide polymorphism	SNP	Automation, easy to establish standard operation, quick, high reliability	Technical difficulty, high chip design costs, require whole genome sequence information	*Perilla frutescens* L. (Shen et al. 2017); *Panax notoginseng*(Burk.)F.H. Chen (Dong et al. 2017); *Bupleurum chinense* DC. (Yang et al. 2026)

With the advancement of molecular biology technologies, DNA molecular markers have become increasingly diversified. Examples include microarray-based DNA fingerprinting methods like Diversity Arrays Technology (DArT) (Kilian et al. 2012), retrotransposon-based methods such as inter-primer binding site (iPBS) (Kalendar et al. 2010), and gene-targeted markers including start codon targeted (SCoT) polymorphism (Collard and Mackill 2009b), conserved DNA-derived polymorphism (CDDP) (Collard and Mackill 2009a), and conserved region amplification polymorphism (CoRAP) (Wang et al. 2009). Compared to other markers, SCoT markers are favored for their simplicity, cost-effectiveness, good reproducibility, high polymorphism, and the advantage that primer design does not require prior genomic sequence information, which has led to their extensive application in medicinal plants. Furthermore, sequence characterized amplified region (SCAR) markers based on SCoT are also employed for species identification, linkage map construction, trait association analysis, and new variety development. SCoT markers provide distinct advantages for genetic and genomic research, as well as for the germplasm improvement across a diverse array of plant species (Rai 2023). For instance, Mulpuri et al. (Mulpuri et al. 2013) utilized 36 SCoT primers to evaluate 48 accessions of *Jatropha curcas* germplasm sourced from various countries. The polymorphic SCoT markers identified were subsequently converted into SCAR markers, which effectively differentiated between toxic and non-toxic strains of *J. curcas*. In another application, Gao et al. (Gao et al. 2023) optimized the SCoT molecular marker reaction system, revealing genetic diversity within 10 wild *Dendrobium officinale* germplasm resources native to Guangxi. Similarly, Wang et al. (Wang et al. 2023b) used SCoT-based cluster analysis to categorize 22 *Ziziphus jujuba var. spinosa* cultivars, providing a basis for germplasm identification and utilization.

By screening superior individual plants in combination with identified molecular markers, researchers can select markers or marker combinations that exhibit significant associations based on correlation analysis, thereby facilitating their application in MAS. For instance, building on the construction of a high-density genetic linkage map and localized QTLs for key traits in *Eucommia ulmoides*, Zhao et al. (Zhao et al. 2017) identified six molecular markers or marker combinations with high detection efficiency. Concurrently, through correlation analysis of phenotypic traits in elite individuals across different age classes (4 to 6 years), they determined that early-stage phenotypic selection is optimal when plants reach 6 years of age. These findings led to the establishment of a MAS system for *E. ulmoides*, offering a novel scientific approach to accelerate the breeding process for this species. *FL-3.2* is a major locus significantly associated with fruit length, width, and shape. Building on this finding, Wang et al. (Wang et al. 2025b) developed an F_2_ population of *Capsicum annuum* L. from two recombinant inbred lines carrying *FL-3.2*^*AA*^ but differing in fruit length, enabling fine mapping of a new minor-effect locus, *FL-10.1*. Unlike *FL-3.2*, *FL-10.1* specifically regulates fruit length without affecting width, highlighting its value for targeted breeding. These findings facilitate the exploration of novel genes regulating key traits and provide deeper insights into the genetic linkages underlying complex traits.

#### Sequencing technology accelerates the identification of genetic information

The development of sequencing technology has significantly accelerated advancements in medicinal plant breeding. Previously, while some genes associated with key traits were identified, most markers were not effectively applicable for MAS. The primary obstacle was that the development of these markers heavily depended on specific populations, making them applicable only to genotypically matched materials (Guo et al. 2023). Gene sequencing can precisely resolve the composition and arrangement of nucleotides (A, T, C, G) at the single-nucleotide level, as well as effectively capture complex structural variations at the chromosomal level (such as large-scale deletions, insertions, or translocations), rather than merely distinguishing between the presence or absence of bands. Rapid advancements in sequencing technology coupled with declining costs have facilitated the extensive detection of genetic variation, thereby enabling breeders to identify more marker-trait association (MTA) loci. This wealth of data enhances the application of MAS, thereby facilitating genomics-assisted selection (GAS). Genome-wide association studies (GWAS) identify genomic loci associated with phenotypic traits by analyzing the significance of associations between genome-wide genetic markers and phenotypic variation in large-scale samples, typically sourced from natural germplasm collections, thereby elucidating the genetic basis of traits at the population level (Du et al. 2025).

Sequencing technologies have substantially advanced variety improvement and functional gene identification in medicinal plants. For instance, Shen et al. (Shen et al. 2017) identified 30 non-synonymous mutation SNP markers through whole-genome sequencing to select superior *Perilla frutescens* L. varieties, and successfully developed the high-yielding, disease-resistant, and drought-tolerant cultivar named “Zhongyan Feisu No. 1”. Dong et al. (Dong et al. 2017) utilized restriction site-associated DNA sequencing (RAD-Seq) combined with PCR technology to screen for associated SNP loci within a disease-resistant population of *Panax notoginseng*. By leveraging these loci to eliminate individuals with non-target traits, the researchers successfully isolated and purified the disease-resistant population, thereby enhancing breeding efficiency and developing the first disease-resistant variety of *P. notoginseng*, “Miaoxiang Kangqi No. 1”. Wang et al. (Wang et al. 2025a) reported a haplotype-resolved genome assembly and annotation for *Areca catechu* L. They identified two tandem genes, *AcGNMT1* and *AcGNMT2*, that mediate the conversion of guvacine to arecoline. These findings provide a high-quality genomic resource and offer insights into arecoline biosynthesis. Liu et al. (Liu et al. 2026) identified the gene *GeSWEET14* in *Gastrodia elata* through single-stem/multi-stem (SS/MS) transcriptome screening, and performed functional analysis on transgenic lines. They reveal that this gene enhances the formation and yield of vegetative propagation corms (VPCs) in *G. elata* by promoting the biosynthesis of polysaccharides and starch. Reynolds et al. (Reynolds et al. 2026) generated a chromosome-scale assembly of the *Withania somnifera* (ashwagandha) genome and identified two withanolide biosynthetic gene clusters exhibiting tissue-specific expression patterns. Through metabolic engineering in yeast, complemented by heterologous expression in *Nicotiana benthamiana* and virus-induced gene silencing in *W. somnifera*, they elucidated the withanolide biosynthetic pathway (Reynolds et al. 2026). Collectively, these sequencing efforts provide a solid foundation for the discovery of functional genes and the improvement of germplasm resources.

Molecular marker-assisted breeding is a crucial component of molecular breeding in medicinal plants. At present, aided by high-throughput sequencing and bioinformatics analysis, breeders can precisely identify key loci controlling target traits from a massive volume of nucleotides, followed by cloning and functional validation. Subsequently, these key loci are converted into specific molecular markers, enabling rapid screening of breeding populations via MAS. Although the widespread application across most medicinal plants is currently progressing through the foundational stages of marker validation, the successful development of superior varieties in a few key species highlights the transformative power of this approach. Undoubtedly, molecular marker-assisted breeding not only significantly enhances the accuracy and efficiency of identifying desirable traits, but the key targets identified also lay a solid foundation for subsequent gene function elucidation and genetic engineering.

### Genetic engineering breeding

**Fig. 2:**
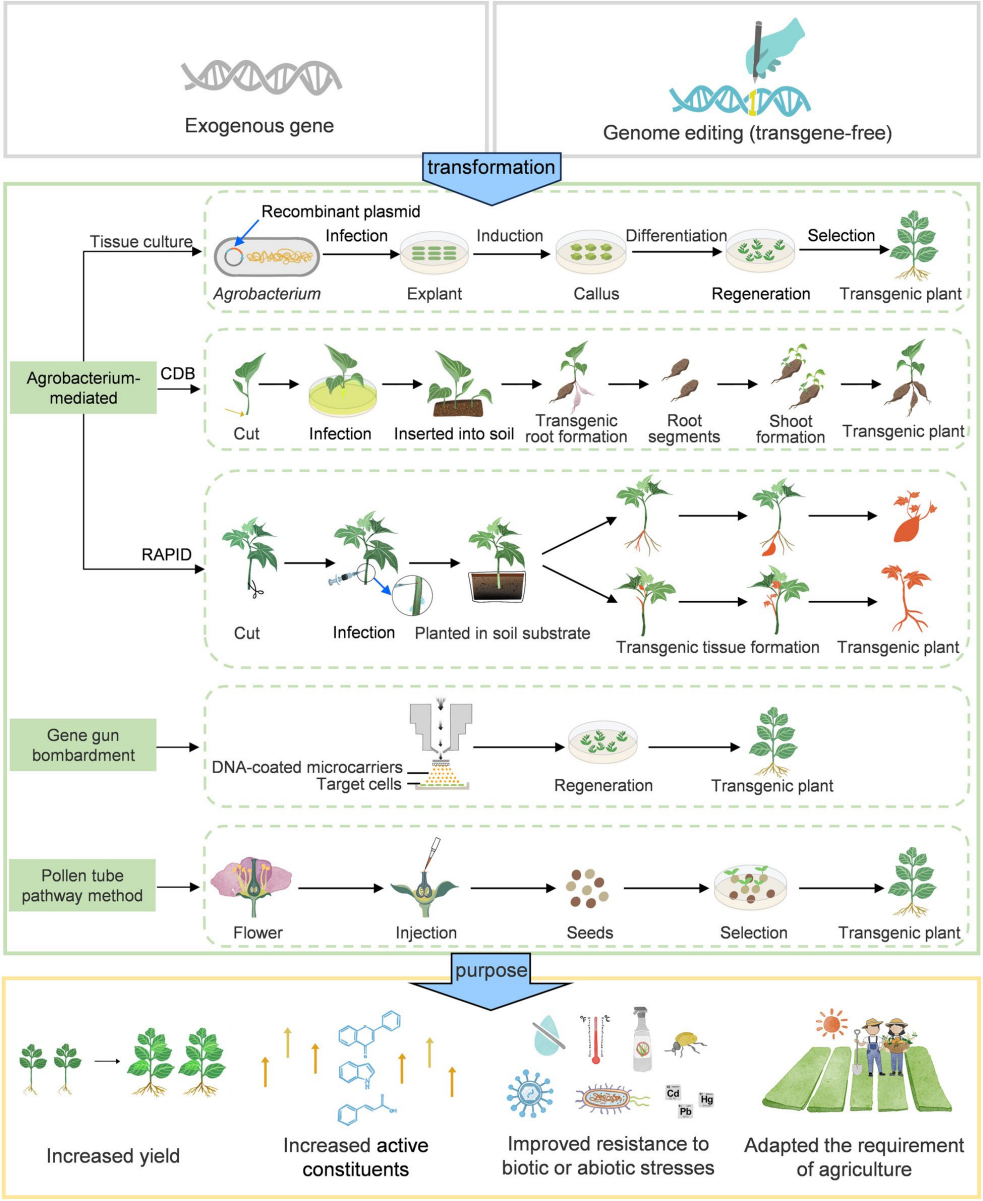
Schematic diagram of the general strategies for genetic engineering breeding in medicinal plants

As a key strategy in plant breeding, genetic engineering utilizes the expression of exogenous DNA to endow recipient plants with desirable characteristics (Chen et al. 2019). This technology holds considerable potential and broad application prospects in boosting active ingredient content, breeding stress-resistant varieties, and optimizing resource utilization (Fig. 2). Beyond transforming known genes for targeted breeding, genetic engineering facilitates the elucidation of unknown gene functions by generating loss- or gain-of-function mutants. Breeding medicinal plants through genetic engineering relies on two critical steps: first, the acquisition and functional validation of genes controlling target traits (such as active constituent accumulation, stress resistance, or growth and development); and second, the establishment of an efficient genetic transformation system to introduce target genes into recipient tissues and induce the regeneration of whole plants (Wu et al. 2020). Consequently, accurate gene characterization and the robust establishment of transformation systems serve as the fundamental prerequisites for genetic engineering breeding in medicinal plants.

#### The methods of genetic transformation

Genetic engineering breeding has developed alongside the establishment of genetic transformation systems (Chen et al. 2019). Currently, the primary methods of genetic transformation include *Agrobacterium*-mediated transformation, gene gun bombardment, pollen tube pathway method, polyethylene glycol (PEG)-mediated method, virus-mediated method, nanoparticle-mediated method, and electroporation method. Among these techniques, *Agrobacterium*-mediated transformation, gene gun bombardment, and pollen tube pathway method are the most frequently employed for the genetic improvement of medicinal plants. Table 2 summarizes the advantages and disadvantages of these common genetic transformation methods as applied to medicinal plants.

**Table 2:** Advantages and disadvantages of common genetic transformation methods in breeding of medicinal plants

Method	Advantages	Disadvantages
*Agrobacterium*-mediated transformation	Easy to transform large pieces of DNA, high efficiency, stable transformation	Time-consuming, constrained by the type of species and explant
Gene gun bombardment	Not limited to plant tissue or cell type, easy to operate, quick	Prone to tissue damage, difficult to achieve stable genetic transformation, high cost, transferring large fragments of DNA can easily lead to integration confusion, prone to gene silencing
Pollen tube pathway method	Without tissue culture or in vitro regeneration	Low transformation efficiency, constrained by natural flowering period
PEG-mediated method	Technical simplicity, low cost	Difficult to achieve stable genetic transformation
Virus-mediated method	Higher rate of conversion	High species specificity, difficult to achieve stable genetic transformation
Nanoparticle-mediated method	Protecting nucleic acids, less pollution, cell wall crossing without external force, low toxicity and low side effects	High cost, difficult to achieve stable genetic transformation
Electroporation method	Ability to transform entire cells and tissues, low cost	Require removal of the cell wall, limited to in vitro suspension systems, prone to tissue damage

*Agrobacterium*-mediated genetic transformation utilizes *Agrobacterium* to deliver T-DNA-based gene expression vectors into plants for genetic modification (Pollari 2024). The commonly used species include *Agrobacterium tumefaciens* and *Agrobacterium rhizogenes*, which harbor the tumor-inducing (Ti) plasmid and the hairy root-inducing (Ri) plasmid, respectively (Gelvin 2017). By modifying these plasmids, the target gene along with the T-DNA region is transferred and integrated into the plant genome (Su et al. 2023). Subsequently, transgenic plants can be successfully regenerated from callus tissue via tissue culture techniques.

In the field of medicinal plants, Agrobacterium-mediated genetic transformation is widely used to enhance the yield of active compounds and improve tolerance to abiotic and biotic stresses, such as drought, salinity, flooding, and disease or pest resistance. The utilization of *A. tumefaciens* facilitates the induction of transgenic shoots for plant regeneration. For example, Meng et al. (Meng et al. 2023) achieved co-expression of the *GR79 EPSPS* and *pGAT* genes in *Medicago sativa* L. through *Agrobacterium*-mediated transformation. The generated genetically modified lines can withstand glyphosate concentration ten times higher than the commercial recommendation, coupled with a significant reduction in glyphosate residues. Xu et al. (Xu et al. 2023) integrated the *LasLYS1* and *LasLYS2* genes from *Candidatus* Liberibacter asiaticus (*CaLas*) into Carrizo citrange via *Agrobacterium*-mediated transformation, successfully developing a citrus variety resistant to Huanglongbing disease. Dong et al. (Dong et al. 2024) employed *Agrobacterium*-mediated transformation to edit and silence the *TLR3* gene in *Artemisia annua*, leading to a line with significantly elevated artemisinin content. Using this transformation method, Gupta et al. (Gupta et al. 2022) enhanced pyrethrin (a natural insecticide) production and improved insect resistance in *Tagetes erecta* by overexpressing the *chrysanthemyl diphosphate synthase* (CDS) gene under the CaMV35S promoter. Yan et al. (Yan et al. 2025) generated *CmACS6* overexpression and disruption lines in *Chrysanthemum morifolium*. They found that overexpression lines exhibited increased ethylene production and reduced waterlogging tolerance, whereas disruption lines displayed the opposite phenotype, indicating that *CmACS6* negatively regulates waterlogging tolerance in *C. morifolium*. In contrast, *A. rhizogenes* is employed to induce hairy roots, which serve as valuable platforms for producing targeted secondary metabolites. For example, Xiao et al. (Xiao et al. 2011) achieved notable increases in rosmarinic acid and lithospermic acid B accumulation in *Salvia miltiorrhiza* hairy roots by co-expressing *tat/hppr* genes, reaching levels 4.3-fold and 3.2-fold higher than the wild type, respectively. In another study, Xiao et al. (Xiao et al. 2020) overexpressed the *IiWRKY34* transcription factor in *Isatis indigotica* hairy roots, markedly increasing the content of bioactive lignans, such as lariciresinol, while enhancing tolerance to salt and drought stress. Jiang et al. (Jiang et al. 2026) established an optimized *Agrobacterium rhizogenes*-mediated transformation system to generate *Glycyrrhiza glabra* hairy root lines overexpressing *GgIFR*. These lines achieved a 44-fold increase in glabridin content, reaching levels comparable to those in 4-year-old wild roots. Gong et al. (Gong et al. 2012) co-transformed the *orca3* and *g10h* genes into *Catharanthus roseus* hairy roots, establishing a rapid propagation system for regenerating plantlets and resulting in a new line with enhanced alkaloid production.

Conventional *Agrobacterium*-mediated transformation typically requires intricate tissue culture protocols that are both time-consuming and prone to variation depending on the plant species and genotype. However, recent research has introduced innovative methodologies to streamline these procedures. For example, Cao et al. (Cao et al. 2023) reported a simplified delivery system known as the “cut–dip–budding” (CDB) system, which involves infecting the stem-root junction with *A. rhizogenes*. The CDB system successfully facilitated the transformation of a diverse range of plants, including herbaceous species such as *Taraxacum kok-saghyz* and *Coronilla varia*, tuberous root plants like *Ipomoea batatas*, and woody plants including *Clerodendrum chinense*, *Aralia elata,* and *Ailanthus altissima* (Cao et al. 2023). Similarly, Mei et al. (Mei et al. 2024) developed a method designated “regenerative activity-dependent in planta injection delivery” (RAPID). This approach involves the direct injection of *A. tumefaciens* into meristematic tissues, where transformation occurs in nascent tissues followed by vegetative propagation to obtain stable transgenic plants. This method has proven effective for plants with strong regenerative capabilities, such as *Ipomoea pes-caprae*. A significant advantage of these methods is that they bypass traditional tissue culture steps and stringent axenic requirements, thereby improving workflow and enhancing transformation efficiency.

Gene gun bombardment, also known as particle bombardment, is a physical method for directly introducing exogenous DNA into the plant genome (Paszkowski et al. 1984; Sanford 2000). This technique is not restricted by the type of recipient material; consequently, cells, callus, immature embryos, and various organs can all serve as targets for transformation. The procedure involves coating the target DNA onto the surface of gold or tungsten particles, which act as microcarriers carrying DNA. These microcarriers are then propelled at high velocity into recipient cells by the force of a gunpowder discharge or a pressurized helium stream, facilitating the entry of the DNA into the cellular interior (Altpeter et al. 2005; Anami et al. 2013). Once inside, the DNA can integrate into the plant’s chromosomes, leading to the regeneration of transformed plants. Gene gun bombardment has been successfully applied to improving traits in various medicinal plants. For instance, Mao et al. (Mao et al. 2008) utilized gene gun bombardment to introduce the rice chitinase gene (*RCH10*) and the alfalfa β−1,3-glucanase gene (*AGLU*) into *Atractylodes macrocephala*, generating transgenic plants with enhanced resistance to *Rhizoctonia solani*. Similarly, Chen et al. (Chen et al. 2016) employed this method to transfer the *Bt* insecticidal gene *Cry2Aa2* into *Brassica alboglabra* L. H. Bailey. Phenotypic assessments revealed that T0 generation of these transgenic plants displayed greater resistance to *Mamestra brassicae* larvae compared to non-transgenic controls. Furthermore, Yang et al. (Yang et al. 2010) utilized gene gun transformation to introduce the drought and salt tolerance gene *lea3* from *Hordeum vulgare* L. into the protocorm-like bodies of *Dendrobium candidum* Wall.ex Lindl, yielding transgenic plants with enhanced salt stress tolerance.

The pollen tube pathway method introduces exogenous DNA into the ovary through injection or infiltration. The DNA is subsequently transported along pollen tube channels into the embryo sac, facilitating the integration of exogenous DNA into the recipient genome. This integration primarily occurs through the transformation of cells lacking mature cell walls, such as egg cells, zygotes, and early embryo cells (Zhou 1989; Ali et al. 2015). This technique is applicable to both monocotyledonous and dicotyledonous plants, encompassing a diverse range of flowering species. Establishing effective experimental protocols requires careful consideration of specific floral structures and the associated pollination and fertilization processes (Zhou 1989). It offers distinct advantages by circumventing the need for tissue regeneration and avoiding genotypic dependencies or culture-induced variations (Hu and Wang 1999). Although limited by the natural flowering window and transformation efficiency, the method has seen successful application in medicinal plants. For instance, Zainudin et al. (Zainudin et al. 2018) selected the *hptII* marker gene and the *gus* reporter gene to establish a pollen tube-mediated transformation system in *Jatropha curcas* L, with transformation efficiencies ranging from 1.5% to 16.7% across three genotypes. Fu et al. (Fu et al. 2025b) employed *AtPAP1* as a marker gene to establish a stable pollen tube-mediated transformation system in *Primula forbesii*, achieving an efficiency of 5.33%. Liu et al. (Liu et al. 2026) used the pollen tube pathway method to transform the gene *GeSWEET14*, achieving a maximum transformation rate of 41.17% and obtaining *G. elata* overexpression lines with increased vegetative propagation corm yield. These successes contribute to the ongoing improvement of these plant varieties.

Other transformation techniques, such as electroporation, PEG-mediated method, virus-mediated method, and nanoparticle-mediated method, have also been explored in medicinal plants. For instance, Wójcik et al. (Wójcik and Rybczyński 2015) utilized electroporation to introduce plasmids carrying the *nptII* and *bar* genes into embryogenic protoplasts of *Gentiana kurroo*. Hu et al. (Hu et al. 2024a) employed a PEG-mediated method to express GFP in protoplasts of *E. ulmoides*, establishing a system for protoplast isolation and transient transformation in this species. Jin et al. (Jin et al. 2023) adopted a virus-mediated method using bamboo mosaic virus (BaMV) to express the exogenous genes *EGFP* and *RUBY* in *Dendrocalamus latiflorus* Munro, while successfully overexpressing the endogenous bamboo genes *ACE1* and *DEC1*. Farooq et al. (Farooq et al. 2022) achieved the transformation of the *GFP* gene in *Abelmoschus esculentus* using green iron nanoparticles. Currently, these methods are predominantly used for transient expression studies; however, they provide significant value in elucidating gene function and exploring genetic transformation systems within medicinal plants.

#### Precise regulation via gene editing technology

Traditional transgenic techniques primarily focus on the introduction of functional exogenous gene fragments into organisms, with integration occurring at random genomic loci (Du et al. 2025). This random insertion poses a risk of disrupting or silencing essential functional genes, potentially hindering plant development. In contrast, gene editing technology offers a superior strategy for precise DNA insertion at specific genomic sites (Dong and Ronald 2021). This technology enables targeted gene modifications, allowing for the precise insertion, deletion, or substitution of bases at defined positions within the DNA sequence to generate desired mutants (Lu et al. 2018). Furthermore, genome editing extends to the fine-tuning of gene expression by modifying regulatory elements, including promoters and untranslated regions (UTRs), thereby holding great potential for plant improvement. Notably, although gene editing involves the initial delivery of exogenous constructs, these sequences are generally unlinked to the target editing sites and can be subsequently eliminated through genetic segregation via backcrossing or selfing. This process ultimately yields edited plants that are devoid of exogenous DNA (transgene-free), representing a significant advantage of this technology (Wu et al. 2020).

Gene editing technologies are generally categorized into three generations: Zinc Finger Nucleases (ZFNs), Transcription Activator-Like Effector Nucleases (TALENs), and the Clustered Regularly Interspaced Short Palindromic Repeats (CRISPR)/Cas system. Sequence-specific nucleases generate double-strand breaks (DSBs) at defined genomic loci for targeted editing. The ensuing breaks are subsequently repaired by the donor-dependent homology-directed repair (HDR) pathway or the non-homologous end joining (NHEJ) pathway (Collonnier et al. 2017). Early systems, such as ZFNs and TALENs, were constrained by low transfection efficiency, complex design requirements, and a limited capability for multiplex gene editing (Chen et al. 2026). The CRISPR/Cas system exhibits superior editing efficiency, simpler design, and greater operational ease compared to earlier technologies, alongside being more cost-effective (Hu et al. 2016, Janik et al. 2020). These advantages have established it as the predominant genome editing technology to date. CRISPR/Cas systems are broadly classified into two classes: class I (encompassing types I, III, and IV) and class II (encompassing types II, V, and VI) (Chen et al. 2026). The CRISPR/Cas9 system, one of the most prevalent gene editing systems, is classified as the type II system (Long et al. 2023).

The CRISPR/Cas9 system serves as a powerful tool in medicinal plants for validating gene function and breeding cultivars with superior traits. For instance, Alagoz et al. (Alagoz et al. 2016) employed CRISPR/Cas9 to knock out the *4′OMT2* gene in *Papaver somniferum* L., significantly reducing the biosynthesis of benzylisoquinoline alkaloids (BIAs) and revealing a novel alkaloid. Zeng et al. (Zeng et al. 2021) used CRISPR/Cas9 to disrupt the hyoscyamine 6*β*-hydroxylase (*AbH6H*) gene in *Atropa belladonna* L. This modification resulted in plants with elevated hyoscyamine levels and the absence of its derivatives, anisodamine and scopolamine. In *Pinellia ternata*, Zhang et al. (Zhang et al. 2021) knocked out the stearic acid desaturase gene (*PtSAD*), which is associated with the thermal stress response; this significantly increased the proportion of saturated fatty acids, conferring heat tolerance. Zhou et al. (Zhou et al. 2024) applied CRISPR/Cas9 to generate single and double mutants of *Sm*CYP98A75 and *Sm*CYP98A14 in the hairy roots of *S. miltiorrhiza*. These mutants demonstrated a significant reduction in active components, such as rosmarinic acid, danshensu, and salvianolic acid B. This discovery confirmed the functional roles of *Sm*CYP98A75 and *Sm*CYP98A14 and laid the groundwork for future breeding initiatives. Beyond CRISPR/Cas9, various gene editing technologies are continuously emerging, such as CRISPR/Cas12a (Zetsche et al. 2015), CRISPR/Cas12b (Shmakov et al. 2015), prime editors (PEs) (Anzalone et al. 2019), glycosylase base editors (GBEs) (Zhao et al. 2021), the TnpB system (Karvelis et al. 2021), and the CyDENT system (Hu et al. 2024b). Several of these tools have already been applied to medicinal plants. For instance, Lv et al. (Lv et al. 2024) successfully utilized a modified TnpB system for gene editing in five distinct medicinal species: *A. annua*, *S. miltiorrhiza*, *Scutellaria baicalensis*, *I. indigotica*, and *Codonopsis pilosula*. Yu et al. (Yu et al. 2026) developed a prime editor designated MediPlant-NEPE for medicinal plants, based on an N-terminal M-MLV RT fused to Cas9 nickase plus epegRNA prime editor (NEPE) (Zhong et al. 2024). This system successfully performed precise editing of key metabolic genes in *S. miltiorrhiza* and *Fagopyrum dibotrys*, significantly increasing the content of target secondary metabolites such as cryptotanshinone, tanshinone IIA, rosmarinic acid, salvianolic acid B, and rutin. These achievements demonstrate the expanding potential of gene editing technologies in medicinal plant research.

Undeniably, genetic engineering has been instrumental in elucidating gene functions and unraveling secondary metabolic regulatory networks in medicinal plants. However, its practical application in medicinal plant breeding remains in its infancy. Most research is still confined to the functional validation of key enzymes and transcription factors, with limited progress in directly engineering new germplasm. This slow progress is further compounded by stringent regulatory frameworks and complex biosafety legislation, which severely restrict the commercialization and field trials of genetically modified medicinal plants. To overcome the persistent bottlenecks in genetic transformation and navigate these legal hurdles, it is imperative to tailor and optimize technical strategies to the specific biological characteristics of each medicinal plant—particularly by adopting transgene-free gene editing to meet compliance standards. Ultimately, this paves the way for transitioning from basic functional validation to the creation of novel, field-ready germplasm in medicinal plants.

### Molecular design breeding

**Fig. 3:**
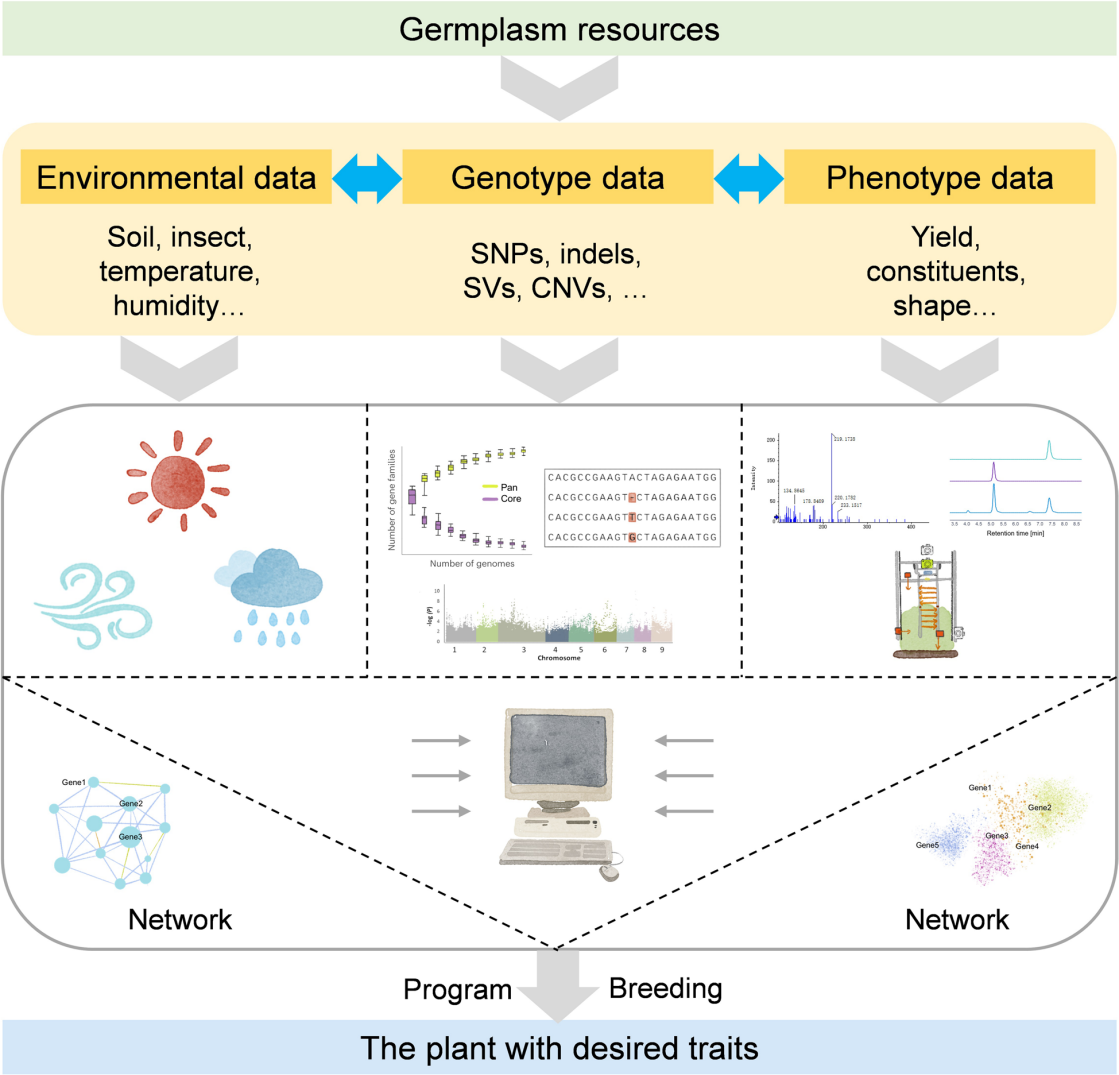
Schematic diagram of the research strategies for molecular design breeding

Molecular design breeding aims to leverage computational technologies to integrate biological big data (including genomic, transcriptomic, proteomic, metabolomic, and environmental data) to simulate, screen, and optimize genetic, environmental, and growth factors. A prerequisite for this is to comprehensively dissect key genes and their regulatory networks underlying target traits, and to acquire high-quality, multi-dimensional datasets that encompass genotypes, phenotypes, envirotypes, and their interactions. Building upon this foundation, breeding models can be trained and optimized by integrating these rich datasets to predict the performance of diverse parental combinations under specific breeding objectives. This facilitates the selection of superior parents and guides targeted screening strategies. By translating these predictive and design strategies into practice via breeding technologies such as hybridization, MAS, genome editing, and synthetic biology, breeders can rapidly develop elite germplasm. Given the tremendous species diversity and specific breeding requirements related to secondary metabolism of medicinal plants, molecular design breeding will undoubtedly unlock unprecedented opportunities for the creation of high-quality varieties (Fig. 3).

#### Integrated management of genomic information

The proliferation of Next-Generation Sequencing has enabled the decoding of genomes for many medicinal plants, including *Lonicera japonica*, *S. miltiorrhiza*, and *A. annua*, resulting in substantial genomic datasets. Building on this foundation, the integration of genomic data with other multi-omics data can assist researchers in elucidating growth and developmental patterns, deciphering secondary metabolic pathways, and identifying key functional genes in medicinal plants. These insights subsequently serve to advance breeding research. Currently, several specialized genome databases and comprehensive repositories for medicinal plants have been established, including the Medicinal Plants multi-Omics Database (MPOD) (He et al. 2022), the Traditional Chinese Medicine Plant Genome (TCMPG) (Meng et al. 2022), the 1 K Medicinal Plant Genome Database (1 K-MPGD) (Su et al. 2022), and MetaDb: a database for metabolites and their regulation in plants with an emphasis on medicinal plants (Gao et al. 2024). These platforms provide valuable resources for researchers engaged in the genetic improvement of medicinal plants.

#### High throughput collection of phenotype data

As genotyping technologies rapidly advance, the collection of phenotypic data has increasingly become the primary bottleneck in genetic studies. High-throughput phenotyping (HTP) comprises a range of methodologies designed for the rapid acquisition and analysis of multidimensional phenotypes, enabling researchers to capture detailed plant performance data across diverse field and natural environments. Technological advancements, particularly in novel sensors, image analysis, robotics, automated equipment, and satellite remote sensing, have significantly augmented the application of HTP. These innovations have transformed phenotypic data collection from labor-intensive manual measurements to high-throughput, high-precision digital acquisition (Zhang et al. 2024a), enabling high-resolution measurement of traits across temporal and spatial scales in both controlled and field environments (Varshney et al. 2021). In recent years, numerous HTP platforms have been established in plant breeding (Araus and Cairns 2014), incorporating diverse technologies such as RGB imaging, normalized difference vegetation index (NDVI) sensors, multispectral and hyperspectral cameras, spectrometers, and light detection and ranging (LiDAR) (Jangra et al. 2021). For instance, Crop3D utilizes multiple imaging sensors within a movable gantry system to quantify 3D plant and leaf architectures as well as leaf temperature (Guo et al. 2018). In *Fritillaria cirrhosa*, a LI-6800 portable photosynthesis system was used to measure photosynthetic parameters (net photosynthetic rate, intercellular CO_2_ concentration, transpiration rate, and stomatal conductance) in the control group and under UV-B treatments, aiming to compare the photosynthetic capacity of wild-type and cultivated plants (Yang et al. 2025). Similarly, to elucidate the mechanisms of altitudinal phenotypic plasticity in *Phlomoides rotata*, continuous monitoring of abiotic variables (air temperature, humidity, soil temperature, moisture, and illuminance) was conducted every 2 h via the JingxunYun platform (Wu et al. 2025). In another approach focusing on hemp achenes, integrating NF 555 spectrophotometry for colorimetric traits (CIE-LAB values) with AP-MALDI-MSI for mapping 55 specific compounds significantly enhanced the accuracy of phenotypic and spatial metabolic characterization (Li et al. 2026). These platforms not only monitor and evaluate growth parameters, ranging from macroscopic traits to internal physiological dynamics, but also provide automated solutions for germplasm resource surveys and environmental data collection (Kim et al. 2020).

#### AI-driven analysis in medicinal plant breeding

AI encompasses a broad spectrum of computational techniques. Machine learning, a crucial subset of AI, utilizes algorithms to learn from data and generate predictions (Zhu et al. 2025); common algorithms include random forest (RF), support vector machine (SVM), k-nearest neighbor (KNN), and neural networks (NN) (Farooq et al. 2024). As a specialized branch of ML, deep learning (DL) employs multi-layer neural networks to automate feature extraction and pattern recognition from complex data for tasks such as classification, prediction, and generation (LeCun et al. 2015). Leveraging these algorithms, researchers can integrate natural language processing and cross-modal data analysis to facilitate DNA sequence feature extraction (e.g., gene recognition, cis-element prediction), germplasm data mining (e.g., germplasm evaluation, environmental adaptability analysis), and agricultural decision support (e.g., planting pattern prediction and disease risk assessment) (Fu et al. 2025a). Currently, while major crops like wheat (Sandhu et al. 2021), rice (Zhang et al. 2022), and soybean (Gill et al. 2022) have advanced into the era of design breeding, medicinal plants remain predominantly in the stage of comprehensive germplasm resource analysis. Meanwhile, AI is actively driving the paradigm shift in medicinal plant breeding towards data-driven, automated, and intelligent practices, thereby significantly accelerating the breeding process. For instance, Azadnia et al. (Azadnia et al. 2022) proposed a DL model comprising a convolutional neural network (CNN) block for feature extraction and a classifier block for feature categorization. This model achieved an accuracy exceeding 99.3% in identifying leaves from five medicinal plants, *Melissa officinalis* L., *Stevia rebaudiana *Bertoni., *Mentha balsamea*, *Aegle marmelos*, and *Ocimum sanctum* L., across three image resolutions (64 × 64, 128 × 128, and 256 × 256 pixels), providing a valuable reference for the development of automated visual recognition systems. Chen et al. (Chen et al. 2023) applied algorithmic models, including random forest, Gaussian naïve Bayes, and artificial neural networks, to analyze images and metabolic profiles of *S. miltiorrhiza* roots. Their findings indicated that total root area is the optimal parameter for predicting biomass, offering practical guidance for root grading and breeding strategies. In a different approach focusing on ecological suitability, Wu et al. (Wu et al. 2024) integrated principal component analysis (PCA), hierarchical cluster analysis (HCA), Mantel tests, path analysis, and random forest to establish an evaluation model for the cultivation of *Panax ginseng* Meyer. They revealed that temperature seasonality and soil available phosphorus significantly influence ginsenoside contents, laying a foundation for the standardized production of high-quality *P. ginseng*. Chen et al. (Chen et al. 2025b) employed AlphaFold3 to predict the protein structures of *GjTPS1* and *GjTPS2*, followed by molecular docking analysis. This structural study identified a specific key residue that dictates substrate selectivity, providing a precise molecular target for the future breeding of superior *Gardenia jasminoides* germplasm.

Medicinal plant breeding is steadily advancing into the era of molecular design. This rapid progression is propelled by the accumulation of biological big data, the dissection of genetic mechanisms, and the establishment of technological systems. The key condition for future design lies in the comprehensive identification of genetic variations and multi-dimensional phenotypes (particularly secondary metabolites), along with the precise characterization of their complex linear or non-linear mappings. To achieve this, the primary objective is to harness multi-omics integration and computational biology to dissect the genetic basis and regulatory networks underlying key quality traits (e.g., active compound accumulation, yield, and stress resistance), thereby uncovering foundational molecular modules for rational design. Subsequently, using whole-genome profiles from core germplasm, researchers can conduct multi-environment and multi-trait predictive modeling to build a breeding system capable of multi-module coupling and assembly. Given the extreme complexity of secondary metabolic pathways in these species, the development of predictive models should follow a progressive trajectory: from single-gene to polygenic, from linear to non-linear, and from species-specific to broad-spectrum. Ultimately, this approach will enable the targeted optimization and assembly of multiple modules at the whole-genome level, providing precise solutions for the intelligent creation of high-quality medicinal materials.

## Challenges

### Research on germplasm resources

Germplasm resources serve as the cornerstone of breeding programs. Medicinal plants are characterized by their immense species diversity and exceptionally rich germplasm reserves, which provide a vast genetic reservoir for molecular breeding. However, to fully translate this rich genetic potential into breeding breakthroughs, current efforts must address several critical gaps: the comprehensive collection and preservation of germplasm; systematic evaluation and identification; and the isolation and purification of genetic materials (Ma and Mo 2017).

Research on medicinal plants should prioritize the systematic collection of germplasm resources encompassing both cultivated and wild species and their phenotypic evaluation conducted over multiple years and across diverse environments. Phenomics platforms can support the precise monitoring of growth dynamics and collection of environmental parameters across different years, developmental stages, and environments to dissect key traits, such as compound yield and stress tolerance, hidden within these diverse resources. The domestication process from wild to cultivated forms is currently underway for numerous medicinal plants, while certain high-demand species (such as *S. miltiorrhiza* and *L. japonica*) have already achieved large-scale cultivation. Despite this rich biological background, fully unlocking the potential of these resources for molecular design breeding is currently hindered by a notable scarcity of tailored genetic populations—such as elite mutants, near-isogenic lines, recombinant inbred lines, high-purity lines, single segment substitution lines, and backcross inbred populations (Ma and Mo 2017). This bottleneck is particularly pronounced in rare and endangered medicinal plants (e.g., *Sinopodophyllum hexandrum*, *Rauvolfia serpentina*), which harbor unique genetic variations but suffer from a critical shortage of accessible germplasm. Sufficient genetic populations are pivotal for bridging the gap between rich raw germplasm and practical molecular breeding, as they are essential for the discovery and validation of functional genes, the elucidation of genetic principles underlying breeding traits, and the in-depth dissection of the genetic basis of heterosis. Consequently, transforming these extensive germplasm resources into well-characterized genetic populations is an imperative first step for advancing medicinal plant molecular breeding.

### Functional genes in medicinal plants remain largely undercharacterized

Although numerous functional genes have been identified in medicinal plants, their number remains limited compared to major crops and model plant species. This is primarily attributed to the diversity of medicinal plants, with only a few species benefiting from in-depth physiological and biochemical research (Chen et al. 2025a). Since secondary metabolites are the core focus of medicinal plant breeding, genes linked to the biosynthesis, regulation, and evolution of secondary metabolites are key targets to be explored. For agronomic traits, although many functional genes have been identified in model plants (such as *Arabidopsis thaliana* and rice), they may exhibit different functions or functional redundancy in medicinal plants. Therefore, there is an urgent need to intensify efforts to identify functional genes in medicinal plants.

The preliminary screening of candidate genes heavily relies on genomics and transcriptomics strategies, yet achieving gap-free assemblies and error-free annotations continues to be elusive. For example, in scaffold-level assemblies of *P. ginseng*, four key genes involved in ginsenoside biosynthesis (*PgHMGS*, *UGT1*, *UGTPg29*, and *UGTPg45*) were absent from both the annotated CDS and the assembly sequences; in *Ginkgo biloba* from chromosome-level assembly, eight genes crucial for flavonoid and ginkgolide biosynthesis (including *GbDXR* and *GbMYBF10*) were missing from the annotated CDS despite being present in the raw genome sequence (Cheng et al. 2025). To address these limitations, future efforts should prioritize the generation of high-quality, gap-free genomes (such as telomere-to-telomere genomes) alongside comprehensive pan-genome and multi-condition transcriptome sequencing, to maximally capture genetic diversity and spatiotemporal gene expression dynamics. Furthermore, fully realizing the potential of these multi-omics resources hinges on integrating robust bioinformatics pipelines and continuously advancing assembly and annotation algorithms.

Based on existing datasets, candidate genes can be identified using strategies such as homology prediction, co-expression analysis, and GWAS. These candidates are then validated through overexpression, gene editing, or gene silencing, combined with metabolomics analysis and phenotypic observations, to elucidate their regulatory roles in specific metabolic pathways. In addition to validation in the native plant, these targets can also be expressed in heterologous hosts (e.g., *Escherichia coli*, *Saccharomyces cerevisiae*, *N. benthamiana*) to reconstruct pathways, facilitating rapid preliminary functional characterization.

### Efficiency and stability of genetic transformation

In molecular breeding, genetic transformation constitutes a pivotal pathway for the development of new cultivars. Current methodologies are heavily reliant on tissue culture, a process that is labor-intensive, time-consuming, costly, and technically demanding. The success of tissue culture is highly contingent upon species and genotype, with plant regeneration capacity and transformation efficiency exhibiting substantial variation across different genetic backgrounds (Altpeter et al. 2016; Mao et al. 2019). Medicinal plants encompass a diverse array of species spanning families such as *Asteraceae*, *Fabaceae*, *Lamiaceae*, *Rosaceae*, *Rutaceae*, and *Apiaceae*, each characterized by distinct biological attributes. While a limited number of species exhibit traits amenable to genetic transformation, the majority (e.g., *Saposhnikovia divaricata*, *P. notoginseng*) require several months to over a year to achieve successful regeneration. Furthermore, the perennial nature of many medicinal species prolongs trait validation and breeding cycles. The protracted culture duration and low transformation efficiency pose significant challenges to the acquisition of transgenic and gene-edited progeny. Currently, the establishment of species-specific genetic transformation systems is required for each medicinal plant, presenting a substantial obstacle to breeding research (Zhang et al. 2024b). In addition to established non-tissue culture systems, recent studies have demonstrated that developmental regulators can enhance plant regeneration and improve transformation efficiency. For instance, the *WOX5* gene (Wang et al. 2022)*,* the *WIND1-ESR1* cascade system (Kshetry et al. 2025), and the REF1 peptide (Yang et al. 2024) have been applied to various plants to augment transformation efficiency. Nevertheless, there remains a critical need for novel methods capable of fundamentally improving transformation efficiency in a manner independent of species or genotype (Yang et al. 2024).

The stable integration and expression of target genes throughout the genetic modification process, along with the potential for unintended consequences, constitute critical issues requiring careful oversight. Key considerations include off-target effects, the presence of residual vector sequences, the stability of gene edits, and the heritability of desired traits across generations. For instance, the persistence of CRISPR constructs can complicate the assessment of phenotypic stability and penetrance, as the continuous activity of Cas enzymes and guide RNAs may lead to ongoing on-target or off-target editing (Gao and Zhao 2014). Despite remarkable advances in gene-editing technologies, achieving precise and efficient modification within complex plant genomes remains a formidable challenge. Editing efficiency is frequently constrained by genomic characteristics inherent to many medicinal plants, such as polyploidy and a high content of repetitive sequences. These obstacles are particularly pronounced when targeting complex metabolic pathways involving multiple regulatory genes (Hou et al. 2025). Furthermore, genetic interventions may inadvertently induce unintended changes in other traits, resulting in unforeseen phenotypic modifications, a phenomenon frequently observed in genetic modification. Field environments differ substantially from controlled laboratory settings, as environmental factors can suppress or alter trait expression. While sequencing technologies facilitate the post hoc detection of off-target effects, continuous refinement of gene-editing tools, comprehensive elucidation of genetic networks, and enhanced bioinformatic prediction remain essential to ensure the stability and predictability of breeding outcomes from the source.

### Issues regarding the construction of breeding design models

The burgeoning volume of phenotypic, genotypic, environmental, and multi-omics data in medicinal plants underscores the need for robust methodologies in data collection, analysis, and integration. Given the biological complexity of these species and the heterogeneity of data standards, ensuring measurement consistency and creating unified datasets is paramount. Unlike the relative abundance and standardization of genomic and transcriptomic datasets, high-quality metabolomic and proteomic data remain scarce, fragmented, and challenging to integrate systematically (Hou et al. 2025). Consequently, innovative tools or methods are required to optimize data definition (via recognition algorithms), ensure accurate measurement (via advanced sensors), facilitate comprehensive collection (integrating micro- and macro-approaches), and accurately differentiate different types and sources of data (via AI algorithms) (Zhu et al. 2025). Although high-quality data underpin accurate phenotypic prediction, challenges such as polygenic linkage, linkage drag, and pleiotropic effects of key genes hinder the precise coordinated regulation of multiple traits (Zhu et al. 2025). Notably, population structure and these genetic factors may lead to spurious genotype–phenotype associations, compromising the inference of gene function. Analyzing phenotypic and multi-omics data for gene combinations governing different traits is essential to elucidate the genetic regulatory networks and interaction effects underlying complex traits such as active constituents, yield, and stress resistance. Building on this, algorithms can be leveraged to simulate the synergistic and antagonistic effects of multiple genes within modules across diverse genetic backgrounds, thereby screening for optimal haplotype combinations or genomic editing targets to achieve targeted regulation of complex traits.

Additionally, the data surge presents significant storage and management challenges. For effective big data utilization, precision and accuracy are crucial, alongside a careful balance between throughput and cost. Establishing comprehensive databases often involves characterizing numerous varieties and generating massive datasets, demanding high bandwidth and substantial storage capacity. Many existing server facilities face limitations regarding space, load-bearing capacity, cooling, and high maintenance costs (EqualOcean Intelligence 2023). However, breeding experts, whose backgrounds typically lie in pharmacology, traditional Chinese medicine, or botany, often lack specialized expertise in bioinformatics and data platform development. Thus, advancing breeding strategies necessitates interdisciplinary integration, requiring either the cultivation of cross-disciplinary talent or close collaboration with experts from computational and analytical domains.

### Biosafety and policy considerations regarding genetically modified and gene-edited varieties

Currently, the evidence regarding the safety of transgenic and gene-editing technologies remains inconclusive. Nevertheless, the deployment of these technologies may entail ecological and biological implications. For instance, transgenic crops engineered for enhanced resilience and competitive ability could pose risks to biodiversity. Genetically modified plants with heightened resistance can adapt to diverse adverse environments; consequently, seed dispersal may facilitate their invasion into adjacent ecological zones, displacing native flora and usurping ecological niches. Additionally, gene flow via cross-pollination with wild relatives may result in transgene escape, posing concomitant threats to indigenous plant populations. The clinical efficacy of medicinal plants is inextricably linked to their active constituents. If transgenic medicinal plants designed to enrich a particular active ingredient escape, they will not only compromise the genetic diversity of wild populations through gene flow, but also potentially shift the chemical profile of traditional medicinal materials. Given that medicinal plants frequently exhibit “multi-component and multi-target” properties, once this compositional balance is disrupted by gene escape, the clinical efficacy rooted in traditional herbal formulation principles would be directly jeopardized. Therefore, a scenario-based application strategy is critical. To maximize breeding benefits, a clear distinction must be made between two categories of genetically modified medicinal materials: those intended solely as “industrial raw materials” for extracting specific active compounds, and those designed for “traditional clinical use” to counteract genetic degeneration and enhance resilience without altering their core metabolite profiles. To mitigate these ecological risks, breeders may employ genetic bioconfinement strategies, such as the insertion of suicide genes or the utilization of male-sterile genotypes. Concurrently, physical management practices, including spatial isolation and the implementation of barriers, should be adopted to minimize genetic exchange between genetically modified crops and wild relatives. Furthermore, specific genetically modified crops harbor exogenous genes designed to express target proteins that may elicit biological reactions upon entering other organisms. This issue is particularly critical for transgenic medicinal plants, as the primary consumers are often patients or individuals in sub-health conditions, for whom the consequences of adverse reactions are substantially more severe. Consequently, it is imperative that breeders conduct rigorous experimental evaluations during the design phase to ensure maximal biosafety.

The societal adoption of genetically modified and gene-edited products faces considerable challenges, particularly regarding policy and regulatory frameworks, which vary significantly across nations. Countries such as the United States, Brazil, and Argentina have adopted proactive policies that promote the cultivation of genetically modified crops and have implemented a relatively lenient regulatory approach for gene-edited crops, often exempting them from the stringent regulations applied to transgenic organisms (Li et al. 2023a). In contrast, the European Union maintains a cautious and conservative stance toward both genetically modified and gene-edited crops, permitting their import while imposing strict regulations on domestic cultivation (Leng et al. 2021, Lin and Wang 2018). In China, where Traditional Chinese Medicine is widely practiced and which is a major consumer of medicinal plants, transgenic varieties of these plants are subject to strict restrictions. The *Good Agricultural Practice for Chinese Crude Drugs* explicitly prohibits the use of genetically modified varieties of traditional Chinese medicinal materials. Furthermore, the utilization of other biotechnologically derived varieties requires enterprises to provide comprehensive risk assessments and experimental data demonstrating the safety, efficacy, and quality of the new varieties. Constrained by stringent regulatory frameworks, genetically engineered medicinal plants rarely progress beyond the laboratory to large-scale field trials. This policy barrier dampens investment from breeders and industry, stalling subsequent development and commercialization. Nevertheless, China has recently begun to approve certain gene-edited and genetically modified crops; for instance, a gene-edited high-oleic soybean variety was approved in 2023, and later that year, multiple companies obtained production and operation licenses for genetically modified corn and soybeans. Compared to food and industrial crops, genetically modified and gene-edited medicinal plant varieties face slower and more challenging adoption.. Given the ongoing lack of consensus regarding the safety of genetically modified varieties, a scientific and rational perspective is essential. For transgenic medicinal plants, beyond conventional assessments of genetic stability and environmental release risks, it is imperative to transcend the traditional “food safety” evaluation paradigm applied to crops. In-depth studies on pharmacodynamics, pharmacology, and toxicology should be conducted to rigorously ensure the safety and efficacy of clinical medication.

## Conclusion and prospect

Amidst the upgrading of the global health industry, medicinal plant breeding faces evolving challenges and new developmental trajectories. The paramount breeding goal is to enhance the biosynthesis of active pharmaceutical ingredients. However, production systems are increasingly constrained by severe pest and disease pressure alongside extreme climatic events (e.g., temperature anomalies, drought, and waterlogging). Furthermore, the demands for streamlining and mechanizing planting operations, genetic degradation caused by long-term artificial domestication, and issues such as continuous cropping obstacles and a reduction in the levels of bioactive compounds, have created a complex crisis. Therefore, developing comprehensive molecular breeding solutions to these intractable problems has become imperative, as it is the fundamental prerequisite for transitioning to simplified and efficient cultivation practices.

Molecular breeding has opened new prospects to overcome these bottlenecks. To effectively harness this technology, its implementation involves critical steps: strengthening germplasm reserves (e.g., broadening genetic variation and establishing core populations), comprehensively dissecting genetic mechanisms (e.g., integrating multi-omics to elucidate trait associations and polyploid evolution), and constructing a standardized innovation pipeline. To break through these specific production and genetic challenges, future efforts must focus on three strategic directions:

First, promote “scenario-based customization” of breeding objectives and reshape germplasm evaluation standards. Given the diversity and highly differentiated applications of active ingredients, future breeding should shift from extensive “generalized improvement” to the creation of “model germplasms” based on end-use scenarios. For instance, in *S. miltiorrhiza*, the water-soluble phenolic acids and lipophilic tanshinones originate from distinct biosynthetic pathways. Therefore, targeted metabolic regulation must be implemented: for single-ingredient targeted drugs (e.g., Salvianolate or Tanshinone injections), “specialized model germplasms” with exclusively high phenolic acid or tanshinone content should be developed, respectively; for clinical decoction pieces, “composite model germplasms” ensuring the balanced accumulation of both component categories are required.

Second, deepen the “cross-coupling” of cutting-edge technologies to overcome bottlenecks in metabolic pathway regulation and directly combat production constraints. Addressing the inherent complexities of medicinal plants—such as polyploidy, large genomes, and extremely complicated secondary metabolic regulation—requires breaking down single-technology barriers. The rapid advent of single-cell and spatial omics enables the mapping of tissue-specific metabolic networks at unprecedented single-cell resolution, substantially enhancing the predictive accuracy of active ingredient synthesis and cracking the “black box”. Simultaneously, synthetic biology serves as a crucial complement to genome editing; leveraging artificial chromosomes, for instance, holds promise for the systematic reconstruction of complex metabolic pathways, enabling the precise targeting of multiple metabolic regulatory nodes and pathway reprogramming. Furthermore, to explicitly address production challenges like severe pest and disease pressures and environmental stress, this multi-omics and gene-editing pipeline must be directed toward creating varieties with broad-spectrum resilience. By systematically pyramiding disease resistance and stress tolerance genes into elite genetic backgrounds, breeding programs can secure a sustainable yield under adverse field conditions.

Third, accelerate the development of an “intelligent closed-loop” breeding system to overcome phenotyping difficulties and curb genetic degradation. The quality traits of medicinal plants (e.g., active ingredient content) cannot be directly judged by the naked eye, which severely limits the efficiency of traditional selection. It is imperative to deeply integrate panoramic genetic dissection, multi-module intelligent design, and environmental control technologies to construct a three-dimensional digital closed loop of “genotype-phenotype-environment”. In this context, plant factories can be utilized to eliminate climate and soil interference. Within this uniform artificial environment, integrating high-throughput phenotyping, metabolite detection, and MAS dramatically accelerates generational turnover and precise screening, thereby exposing the true genetic potential of plants. Ultimately, this shift from experience-based to data-driven selection not only guarantees the efficient identification of superior lines, but also rigorously culls genetically degraded individuals, thereby preserving the sustainable genetic fidelity of elite germplasm.

Overall, driven by the convergence of molecular breeding and multidisciplinary technologies, medicinal plant breeding is no longer a mere extension of crop breeding, but a profound scientific exploration confronting the complexities of secondary metabolism. It is rapidly transcending traditional empirical limitations, fully entering a new era defined by digitalization, automation, and intelligent design. This will provide a scalable theoretical and technological pathway for systematically mitigating quality degradation, and offer sustainable, source-level innovation support for the high-quality development of the global botanical medicine industry.

## Data Availability

No datasets were generated during the current study.

## Abbreviations

MAS: Marker-assisted selection
AI: Artificial intelligence
HRMS: High-resolution mass spectrometry
NMR: Nuclear magnetic resonance
QTL: Quantitative trait loci
RFLP: Restriction fragment length polymorphism
PCR: Polymerase chain reaction
RAPD: Random amplified polymorphic DNA
SRAP: Sequence-related amplification polymorphism
TRAP: Target region amplification polymorphism
AFLP: Amplified fragment length polymorphism
SSR: Simple sequence repeat
ISSR: Inter-simple sequence repeat
CAPS: Cleaved amplified polymorphic sequence
SNP: Single nucleotide polymorphism
MTA: Marker-trait association
GAS: Genomics-assisted selection
GWAS: Genome-wide association studies
RAD-Seq: Restriction site-associated DNA sequencing
SS: Single-stem
MS: Multi-stem
VPCs: Vegetative propagation corms
DArT: Diversity Arrays Technology
ESTs: Expressed sequence tags
iPBS: Inter-primer binding site
SCoT: Start codon targeted
CDDP: Conserved DNA-derived polymorphism
CoRAP: Conserved region amplification polymorphism
SCAR: Sequence characterized amplified region
PEG: Polyethylene glycol
Ti: Tumor-inducing
Ri: Root-inducing
CDB: Cut-dip-budding
RAPID: Regenerative activity-dependent in planta injection delivery
BaMV: Bamboo mosaic virus
UTRs: Untranslated regions
ZFNs: Zinc Finger Nucleases
TALENs: Transcription Activator-Like Effector Nucleases
CRISPR: Clustered Regularly Interspaced Short Palindromic Repeats
DSBs: Double-strand breaks
HDR: Homology-directed repair
NHEJ: Non-homologous end joining
BIAs: Benzylisoquinoline alkaloids
GBEs: Glycosylase base editors
PEs: Prime editors
NEPE: N-terminal M-MLV RT fused to Cas9 nickase plus epegRNA prime editor
HTP: High-throughput phenotyping
LiDAR: Light detection and ranging
MPOD: Medicinal Plants multi-Omics Database
TCMPG: Traditional Chinese Medicine Plant Genome
1 K-MPGD: 1 K Medicinal Plant Genome Database
ML: Machine learning
DL: Deep learning
RF: Random forest
SVM: Support vector machine
KNN: K-nearest neighbor
NN: Neural networks
CNN: Convolutional neural network
PCA: Principal component analysis
HCA: Hierarchical cluster analysis
